# The Future of Native Bees in Australia Under Environmental and Anthropogenic Change

**DOI:** 10.1002/ece3.74363

**Published:** 2026-09-29

**Authors:** Scarlett R. Howard, Kit S. Prendergast

**Affiliations:** ^1^ School of Biological Sciences Monash University Clayton Victoria Australia; ^2^ School of Molecular and Life Sciences Curtin University Bentley Western Australia Australia; ^3^ Centre for Sustainable Agricultural Systems University of Southern Queensland Toowoomba Queensland Australia

**Keywords:** anthropogenic change, Australia, conservation, environmental change, insect decline, native bees, pollinator decline, southern hemisphere

## Abstract

The extent and causes of recent regional and global insect declines have been heavily debated, raising important arguments, primarily concerning a lack of knowledge on insect pollinators and their population trajectories. Bees are considered one of the most important groups of pollinators. Anthropogenic activities such as land‐clearing, agricultural activities, urbanisation, pollutants, pesticide use, non‐native flora and fauna introductions, disruption of natural fire regimes and climate change, among other threats, are having varied impacts on bees. Australia is a biogeographically isolated continent estimated to host over 2000 native bee species, with the vast majority of species found nowhere else in the world. Despite the potential ecological and economic importance of Australian bees, there is a paucity of information regarding their behaviour, distribution, conservation status, pollination abilities and responses to anthropogenic threats. Native bees are economically undervalued in Australia; however, efforts to explore their crop pollination abilities and value to the economy are underway. Here we discuss the past and current research into native Australian bees regarding anthropogenic influences in light of emerging research into pollinator declines. We highlight the importance of studying an island continent with a rich diversity of endemic bees and provide a framework for future research and conservation efforts. The framework includes suggestions for long‐term monitoring of bee behaviour, species richness, abundance and distributions using a variety of methods to capture information across species, locations and seasons. We advocate for continued and enhanced engagement with the public, who are already contributing to community science projects and online repositories. Our framework provides advice for future monitoring, assessment, public engagement and target areas for future research, which can also be implemented at a global level.

## Graphical abstract

Bees are considered one of the most important and abundant pollinators across the globe, yet they are under threat from anthropogenic activities such as land‐clearing, urbanisation, agricultural activities, pesticide use, non‐native flora and fauna introductions, disruption of natural fire regimes, pollution and climate change, among others. Australia is a unique continent as it is biogeographically isolated as an island with many endemic bee species found nowhere else in the world. Our review, highly informed by research in Australia and across the globe, provides a framework for future monitoring, assessment, public engagement, and target areas for research which can be implemented both in Australia and worldwide.

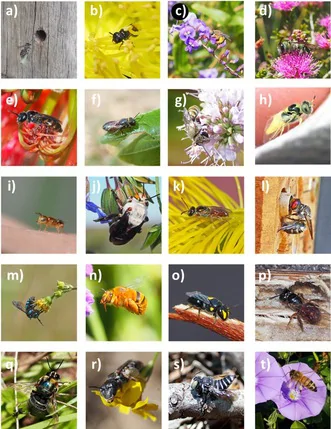

## 1 Introduction

Global and regional declines in pollinating insects have been widely recorded and reported (Goulson 2019; Hallmann et al. 2017; Lister and Garcia 2018; Sánchez‐Bayo and Wyckhuys 2019; Wagner 2020; Wagner et al. 2021). The implicated drivers of these reported declines include climate change, habitat loss, pesticide use, invasive species introductions and a variety of other anthropogenic activities (Baldock 2020; Batley and Hogendoorn 2009; Brown and Paxton 2009; Goulson 2019; Harvey et al. 2020; Lister and Garcia 2018; Owens et al. 2020; Potts et al. 2010; Sánchez‐Bayo and Wyckhuys 2019; Wagner 2020). Whilst there remains debate in terms of the extent of the decline across pollinators and the main driver(s) [e.g., (Cardoso and Leather 2019; Goulson 2019; Hallmann et al. 2017; Lister and Garcia 2018; Sánchez‐Bayo and Wyckhuys 2019; Saunders 2019; Saunders et al. 2019, 2020; Thomas et al. 2019; Wagner 2020)], there is nevertheless a general consensus that we need more information on insect populations. Considering significant insect declines have been observed in long‐term studies in Germany (76% decline over 27 years) (Hallmann et al. 2017) and Puerto Rico (75% and 98% over 35 years) (Lister and Garcia 2018), there is a need to determine the situation in regions with fewer long‐term studies on insect populations, such as Australia (Hoh 2020; Pyke et al. 2023; Sands 2018; Taylor et al. 2018).

Bees are one of the most important and abundant pollinators across the globe (Ballantyne et al. 2017). There are recent reports of managed honeybee colony losses from disease, pests and anthropogenic impacts, which have major impacts for pollination services, with flow on impacts to agriculture, food production and the economy (Gallai et al. 2009; Johnson 2007). Additionally, declines in wild bees have been documented at global, regional and local levels (Bartomeus et al. 2013; Cardoso and Gonçalves 2018; Glaum et al. 2017; Grixti et al. 2009; Jacobson et al. 2018; Koh et al. 2016; Mathiasson and Rehan 2019, 2020; Nooten and Rehan 2020; Pereira et al. 2020; Zattara and Aizen 2021). There have been declines in bees reported from long‐term studies in New England (Mathiasson and Rehan 2020), the Netherlands (Biesmeijer et al. 2006; Scheper et al. 2014), Britain (Biesmeijer et al. 2006; Powney et al. 2019), the United States (Cumberland 2019; Koh et al. 2016) and Brazil (Martins et al. 2013). Many native bees are identified as at risk from land‐clearing and habitat loss/fragmentation, urbanisation, pesticide and insecticide use, climate change, disease, fire and invasive species introductions—including that of the European honeybee (Batley and Hogendoorn 2009; Brown and Paxton 2009; Cardoso and Gonçalves 2018; Glaum et al. 2017; Goulson et al. 2015; Herrera 2020; Mallinger et al. 2017; Mathiasson and Rehan 2020; Owens et al. 2020; Pereira et al. 2020; Potts et al. 2010; Santos et al. 2020). Alarmingly, a recent study reports that the number of global bee species has steadily declined by approximately 25% since the 1990s, and while this could be due to changes in data collection methods and/or reporting, the results may represent a true global decline in bee diversity, which is a cause for concern and action (Zattara and Aizen 2021).

Native wild bees, among other wild insects, are often overlooked in research, pollination, crop production and in economic value (Cardoso et al. 2020; Dunn 2005; Leather et al. 2008; Titley et al. 2017), but recent research has started tackling this issue. Some research suggests that wild bees and insects may even be more effective at agricultural pollination than honeybees in some cases (Karasiński 2018; MacInnis and Forrest 2019; Mallinger and Gratton 2015). A recent study determined that wild pollinators provide a US annual production value of over US$1.5 billion when examining just seven crops (Reilly et al. 2020). In order to create management plans, design effective urban green areas for pollinators, and manage our impact on pollination resources, we need to understand the complex interactions between anthropogenic change and pollination systems.

In Australia there is a significant lack of information surrounding anthropogenic impacts on native bee species (Batley and Hogendoorn 2009; Pyke et al. 2023). While there are some studies looking at species richness, abundance, distribution and flower visits as a result of habitat (Prendergast and Ollerton 2021; Threlfall et al. 2015), floral resources (Threlfall et al. 2015), non‐native honeybee presence (Batley and Hogendoorn 2009; Prendergast, Dixon, and Bateman 2021; Prendergast and Ollerton 2021; Sugden et al. 1996) and climate change (Dew et al. 2019; Santos et al. 2020), little is known concerning how anthropogenic change impacts the behaviour and morphology of Australian native bees. Fifteen years ago, the state of native bees in Australia was reviewed suggesting that the predominant issue with native bee conservation was the shortage of knowledge available (Batley and Hogendoorn 2009). Unfortunately, there is still a paucity of research in this area. Thus, in light of evidence of global, regional and local bee and other insect declines, we revisit the current state of Australian native bees. Our review looks at both past studies, as well as what has been learnt over the past 15 years from national and international research, which can be applied to an Australian context. From the perspective of global and regional insect decline, this review explores the importance of native Australian bees, their environmental requirements, the impacts of different anthropogenic changes and stressors, and how we can plan for the future of bee conservation and research on a continent with a vast range of endemic bee diversity (Houston 2018; Michener 2007, 1965).

Batley and Hogendoorn (2009)'s review of native Australian bees provided a much‐needed foundation for increasing scientific attention to Australia's native bee fauna. Our present review expands upon this past work to identify risks and resources, update the state of the field owing to a major increase in publications on native bees in the past decade, identify what has changed and what has not, and crucially, we provide a road‐map forward for tackling the crises in bee taxonomy, conservation and ecological knowledge. As we will outline below, this research synthesis and framework is much needed because while it is evident there have been some advances in research on Australian native bees, many critical gaps remain which are a barrier to ensuring conservation success into the future.

## 2 The Current State of Bees in Australia

There are an estimated 1760–3000 native bee species in Australia (Australian Biological Resources Study 2020; Batley and Hogendoorn 2009; Dorey et al. 2026; Houston 2018; Walker 2024) comprised of five families including Apidae, Colletidae, Halictidae, Megachilidae and Stenotritidae (Figure 1; Table 1). There are currently 1730 named species. The family Stenotritidae and subfamily Euryglossinae (Colletidae) are endemic to the region (Michener 2007, 1965, 2000), and due to the biogeographical isolation of Australia, the vast majority of species are found nowhere else in the world. There is evidence to suggest that at least 300–500 species of bees are undescribed in Australia [(Batley and Hogendoorn 2009) but see (Dorey et al. 2026)] and new species are still being discovered and described (Leijs et al. 2018; Prendergast and Campbell 2025). There are about 11 (perhaps up to 14) species of eusocial bee in Australia (tribe: Meliponini; genera: *Tetragonula* and *Austroplebeia*), with the vast majority being solitary, and others classified as semi‐social (subsocial; semisocial; quasisocial) (Houston 2018; Michener 2007). Approximately 30% of all bees nest in pre‐existing cavities such as borer holes in wood or hollow plant stems, and whilst some make their own cavities above ground with most bees (about 60%–83%) nesting in the ground (Antoine and Forrest 2021; Dorey 2018) (Table 1).

**FIGURE 1:**
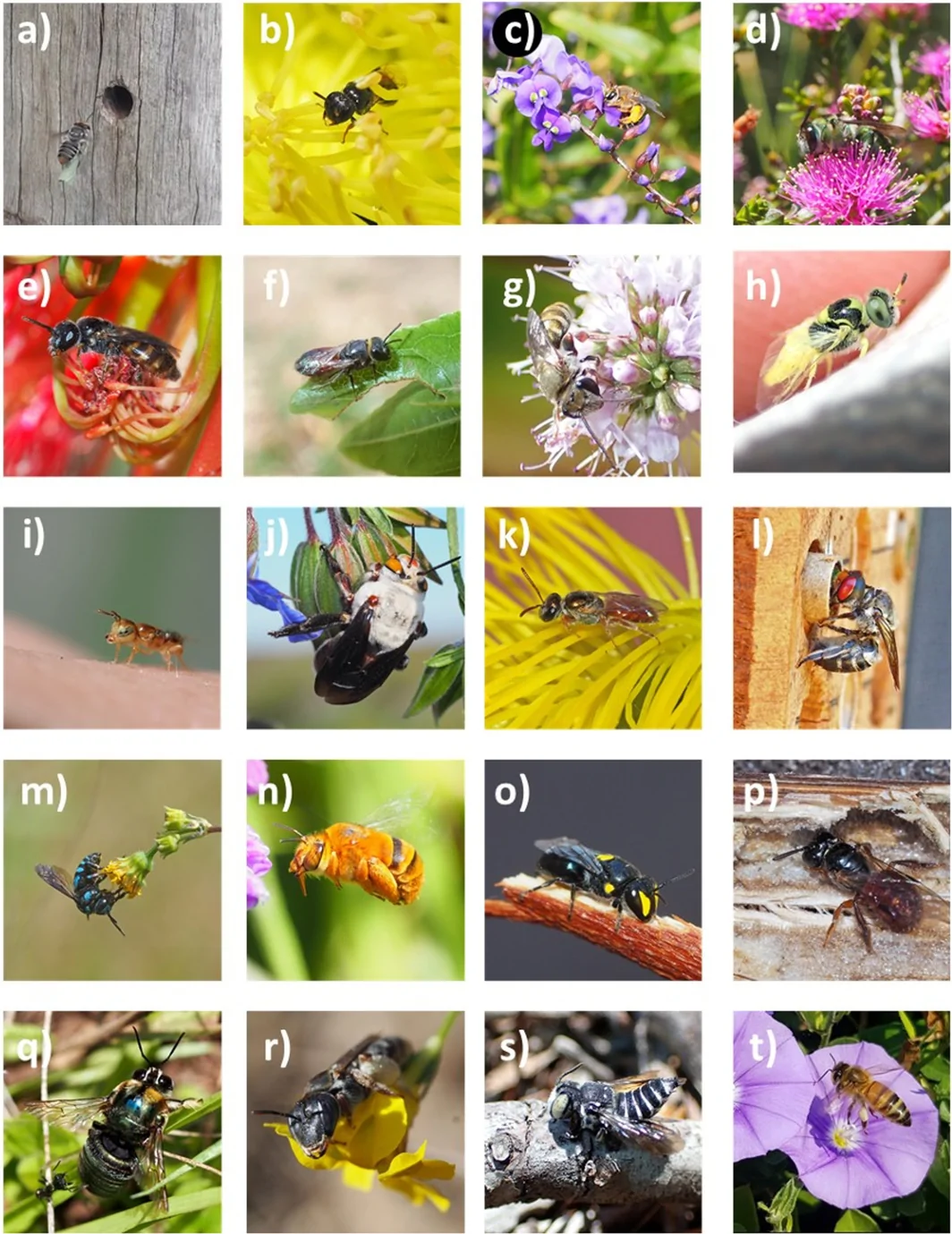
Bee diversity in Australia. Bees shown include (a) *Megachile* spp., (b) *Euhesma* spp., (c) *Trichocolletes* spp., (d) *Ctenocolletes smaragdinus*, (e) *Euhesma tubulifera*, (f) *Euryglossa calaina*, (g) *Lipotriches flavoviridis*, (h) *Xanthesma brachycera*, (i) *Euryglossina mellea*, (j) *Amegilla dawsoni*, (k) *Lasioglossum dotatum*, (l) *Megachile aurifrons*, (m) *Thyreus nitidulus*, (n) *Amegilla bombiformis*, (o) *Hylaeus ruficeps kalamundae*, (p) *Exoneura* spp., (q) *Xylocopa* spp., (r) *Leioproctus douglasiellus*, (s) *Coelioxys froggatti* and (t) *Apis mellifera*. Photos (b) to (s) provided by author K.S.P. Photos (a) and (t) provided by author S.R.H.

**TABLE 1:** Estimates of the current number of native and introduced bee species in Australia by nesting type, sociality, conservation status and taxonomy.

Estimate type	Estimate range	Source/s
Current number of described native bee species (2026)	1730	Australian Biological Resources Study (2020), Walker (2024)
Current estimated number of undescribed species (2026)	300–400; 472	Batley and Hogendoorn (2009), Dorey et al. (2026)
Number of native bee families	5	Australian Biological Resources Study (2020), Walker (2024)
Number of described native bee genera	65	Australian Biological Resources Study (2020), Walker (2024)
Current number of species given legislative conservation listing as a ‘threatened’ status (EPBC Act)	3	Australian Government—Department of Agriculture, Water and the Environment (2020)
Number of introduced bee species	7	Agriculture Victoria (2020), Anderson (2006), Baumann et al. (2016), Hogendoorn and Keller (2011), Houston (2018), Prendergast (2020a), Woodward (1994, 1996)
Number of described native non‐eusocial bees	1719	Australian Biological Resources Study (2020), Walker (2024)
Number of described native eusocial bees	11	Australian Biological Resources Study (2020), Stringer et al. (2012)
Number of described ground nesting native bees	1059	Prendergast (2026)
Number of described above ground nesting native bees	646	Prendergast (2026)
Number of described parasitic native bees	25	Smith and Schwarz (2009)

Three species of Australian native bee have been given an official conservation status at state and federal levels (Australian Government—Department of Agriculture, Water and the Environment 2020), and three additional species are listed on the Western Australia Threatened and Priority Species list (Department of Biodiversity, Conservation and Attractions 2020) (Table 1). *Leioproctus douglasiellus* , *Neopasiphae simplicior* and *Hesperocolletes douglasi* (Colletidae) are listed as critically endangered under the Environment Protection and Biodiversity Conservation Act 1999 (Day et al. 1999). Both *L. douglasiellus* and *N. simplicior* are also classified as ‘fauna that is rare or is likely to become extinct’ under the Western Australian Wildlife Conservation Act 1950 and are managed as ‘endangered’ (according to IUCN criteria) by the Western Australian Government (Department of Sustainability, Environment, Water, Population and Communities 2013; Department of the Environment, Water, Heritage and the Arts 2008). *L. douglasiellus* is found in just three locations within the Perth metropolitan area (Department of Environment and Conservation (DEC) 2009). It occurs in 24.3 km^2^ with an area of occupancy of 0.2 km^2^. It has been collected on two plant species, which are both on the DEC Priority Flora list: *Goodenia filiformis* (Priority 3) and *Anthotium junciforme* (Priority 4) (Department of Environment and Conservation (DEC) 2009). *N. simplicior* has been found at five locations, however recent surveys at two of these locations failed to find the species still present there (Prendergast 2019). *Hesperocolletes douglasi* was listed as ‘presumed extinct’ in 1994, however in 2015 a specimen was found in an isolated woodland remnant in the Southwest Floristic Region of Western Australia (Arnold et al. 2019; Western Australian Government 2018). Further efforts to find other individuals have been unsuccessful and the species is now listed as critically endangered (Arnold et al. 2019; Threatened Species Scientific Committee 2019). Under the Western Australia Threatened and Priority Species list (Department of Biodiversity, Conservation and Attractions 2020), *Glossurocolletes bilobatus* (Priority 2), *Hylaeus globuliferus* (Priority 3), and *Leioproctus contrarius* (Priority 3) are also given a conservation status at state level. The actual number of species that would warrant conservation status is far greater. For example, despite fulfilling criteria for being listed as of conservation concern, *Pharohylaeus lactiferus* , a species rediscovered after not being recorded for over 100 years, remains unlisted (Dorey 2021), as does a *Leioproctus zephyr*, a new highly‐specialised species known from just five locations on the urbanised Swan Coastal Plain, where the other conservation‐listed native bees occur (Prendergast 2022). In addition, based just on loss of habitat from the 2019/2020 bushfires, a further eleven species warrant conservation listing (Dorey et al. 2021).

There are seven species of introduced bee in Australia including the European honeybee ( *Apis mellifera* ), the Asian honeybee ( *A. cerana* ), the European bumblebee ( *Bombus terrestris* ), the African Carder bee ( *Afranthidium repetitum* ) (Baumann et al. 2016; Prendergast 2020a), the European wool carder bee ( *Anthidium manicatum* ) (Agriculture Victoria 2020; Baumann et al. 2016), the Emerald Furrow bee ( *Halictus smaragdulus* ) (Houston 2018) and the Alfalfa Leaf‐cutter bee ( *M. rotundata* ). *Apis mellifera* was intentionally introduced to Australia with European colonisers, whilst the Alfalfa Leaf‐cutter bee ( *M. rotundata* ) was intentionally introduced to South Australia in the 1990s; however, it appears the population did not establish (Anderson 2006; Hogendoorn and Keller 2011; Woodward 1994, 1996).

### 2.1 Importance of Native Bees in Australia

Compared with eusocial managed species such as *A. mellifera* , the economic value of native Australian bees across most crops is currently unknown. Although the foraging repertoire of many species requires further research, records suggest most of the 800+ species of Euryglossinae (Michener 2007), and all 40 species of *Trichocolletes* are oligolectic (i.e., specialise on pollen from just one plant lineage (Batley and Houston 2012)), with oligoleges also represented in Hylaeinae, Colletinae, and Megachilidae (Michener 2007), and restricted foraging preferences even in polylectic species such as *Amegilla dawsoni* (Houston 1991). As such, over half of all species are specialists. Likely due to the long biogeographic isolation of the Australian continent, native bee assemblages overwhelmingly prefer native flora [(Prendergast and Ollerton 2021) but see (Howard and Symonds 2023)], and along with being mainly solitary or semi‐social, they tend to comprise the minority of crop visitors (Hogendoorn et al. 2021). In addition, the Euryglossinae and Hylaeinae bees, which comprise the majority of native bee species in Australia, swallow pollen and lack scopae, making them less effective pollinators (Michener 2007).

Nevertheless, some native bee species do serve as complementary or even better pollinators of crops than managed species (detailed below). When it comes to indigenous Australian flora, pollination services by native bees are essential for many native plants and ecosystems, and therefore have cultural, ecological and economic importance for the wildflower (Beal 1998; Rural Industries Research and Development Corporation (RIRDC) and Karingal Consultants 1997) and bushtucker industries (Cherikoff 2000; Johnston 2002), as well as importance for rare and threatened flora (Gilpin et al. 2014; Prendergast and Campbell 2025). Despite their importance, the pollinators of many native flora remain unknown. In addition, native bees contribute to crop pollination and may have important roles in the future, especially regarding indigenous foods, such as the Kakadu Plum (Territory Natural Resource Management 2017). Unlike *A. mellifera* , numerous native bees such as *Xylocopa*, *Amegilla* and *Lipotriches* species are capable of sonication (Cardinal et al. 2018), and therefore are beneficial for the pollination of tomatoes (Bell et al. 2006; Hogendoorn et al. 2000, 2006, 2007, 2010) and potentially other buzz‐pollinated crops. The eleven described species of Meliponini (‘sugarbag bees’, of which *Tetragonula carbonaria* is the main managed species), like *A. mellifera* , are eusocial and can be managed in colonies, and are used for the pollination of various crops including Macadamias, blueberries, raspberries, lychees and more (Bray 1973; Heard 1999; Heard and Dollin 2000). Globally, stingless bees pollinate over 90 crop species (Heard 1999). The use of two *Amegilla* ‘blue banded bee’ species—the blue‐green blue‐banded ( *Amegilla chlorocyanea* ) and Murray's blue‐banded bee ( *A. murrayensis* )—in lucerne pollination has been explored, and whilst *A. murrayensis* did not appear to pollinate lucerne crops, preliminary experiments showed that *A. chlorocyanea* produced a greater seed yield than honeybees (Hogendoorn 2018). Nine *A. chlorocyanea* individuals foraging in a cage of 100 plants produced a greater yield than 500 *A. mellifera* workers (nucleus hive) (Hogendoorn 2018). A native resin bee, *Megachile nigrovittata* , is also a potential pollinator of lucerne (Hogendoorn and Keller 2012). The yearly contribution of wild pollinators, including native bees and feral honeybees, to lucerne seed production in Australia is worth around $20—25M AUD for dryland lucerne (Hogendoorn 2017), thus understanding and supporting our native bees is of current and future economic importance.

The European honeybee is widely used in Australia to pollinate crops. They can improve fruit quality, yield, size, weight, aesthetic value, commercial value and shelf life (Free 1968a, 1968b; Goodwin 2012; Hughes 1961; Klatt et al. 2014). However, they may compete with native Australian bees for resources (Paini 2004; Paini and Roberts 2005; Prendergast, Dixon, and Bateman 2021; Sugden et al. 1996). Furthermore, *Varroa destructor* , a mite causing mortality and disease, among other impacts, in honeybee hives has recently reached Australian shores (Chapman et al. 2023; Cunningham et al. 2002). The previous estimated economic loss to producers and consumers of bee pollinated crops if *V. destructor* was to establish and spread in Australia ranges from $0.63 billion to $1.31 billion over 30 years (Hafi and Hafi 2012). Thus, it is imperative to explore other options for pollinators in Australia. The first step to future‐proofing our agricultural sector from *V. destructor* is to examine the behaviour, floral preferences, decision‐making and pollination ability of our native bees.

Native Australian bees in the tribe Meliponini have had significant cultural and practical importance to First Nations people for over 65,000 years (Clarkson et al. 2017; Fijn 2014; Halcroft et al. 2013; Perichon et al. 2020; Prendergast, Garcia, et al. 2021). Neparrnga Gumbula, a man of the Yirritja moiety has described his kinship with the Birrkuda (or ‘short‐nosed bee’): ‘My mouth is the entrance to the beehive. My nose is beeswax. My eyes are nuts from the warraga (“cycad palm”). My hairs are the fine roots of the mayku (“paperbark tree”) and the wulu (“white foam”) that they produce in the swamp at Djiliwirri. My head and my knowledge are guku (“honey”) from the wangarr Birrkuda (“Short‐Nosed Bee”). In death, my name is no longer Neparrnga. It is Birrkuda’. (Fijn 2014). First Nations peoples have used the beeswax from native ‘stingless’ or ‘sugarbag’ bees (*Tetragonula* and *Austroplebeia* spp.) to produce rock art for at least 4000 years in Australia (Nelson et al. 1995; Watchman and Jones 2002; Welch 1995), with stingless bee hives as a common motif (Akerman 1979). Honey and wax from these native bees have been used in gifts, diet, art, religion, medicine, children's toys and tool production (Akerman 1979; Welch 1995). Native beeswax has also been used in the production of gum cement for to make weapons, tools, Firesticks, waterproof canoes perform repairs, and for ‘cementing the barbs of spears’ (Powell et al. 2013; Welch 1995), as well as the formation of the mouth piece of didgeridoos (Welch 1995). Sugarbag bees also appear in traditional stories such as ‘Jabreen the Warrior’ of the Kombumerri people (Middleton 2019), the story of a bee woman abducted by a fly man (Bozic and Marshall 1972), the story of the Wawilak sisters searching for honey (Fijn 2014) from the Yolŋu peoples in northeast Arnhem Land (consisting of many different clan groups), and in story of ‘The First Bees’ from northern Gunwinggu (or alternatively spelt Kunwinjku) (Fijn 2014). In Yolŋu culture, many ceremonies involve searching for sugarbag bee nests, which is also a practice linked with key ancestral beings (Fijn 2014). The extensive knowledge regarding native bee ecology, behaviour and nest structure as well as the use of bee products suggest a semi‐domestication of native bees by many First Nations people well before the present time (Perichon et al. 2020).

#### 2.1.1 What Resources Are Important to Native Australian Bees?

Different bees have different resource requirements, and in Australia, more information on what native bees require is needed. Often, plans to support and protect bees focus on providing floral resources; however, bees also depend on non‐floral resources, which are overlooked in many conservation schemes (Requier and Leonhardt 2020). Bees require non‐floral resources such as bare ground to form burrows, cavities to nest in (wood or hollow stems), and access to nesting material such as leaves, resin and many other resources (Prendergast 2018) (Figure 2).

**FIGURE 2:**
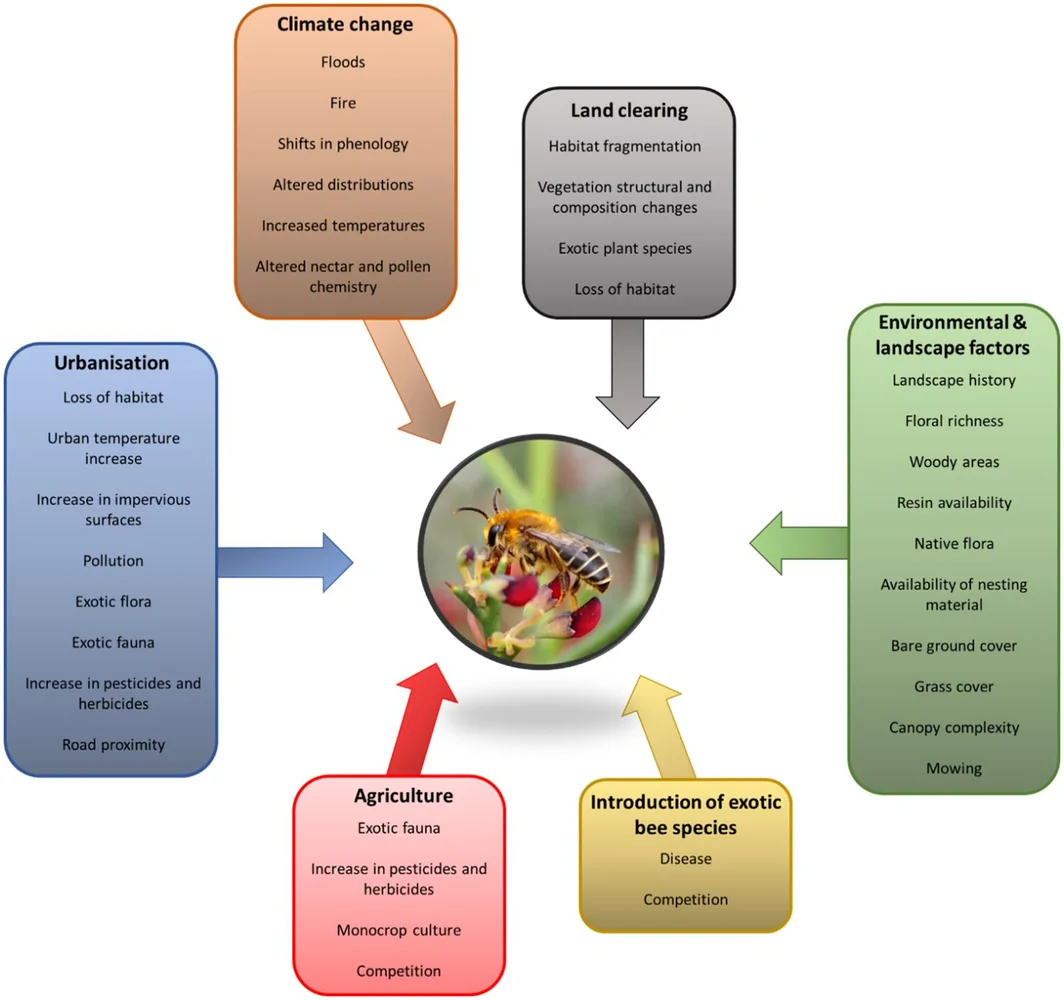
Major anthropogenic and environmental changes known to impact bees and the potential influences of these threats on native bee populations. Image depicts a male *Trichocolletes*. Image credit: author K. S. P.

Habitat suitability for bees in Australia and globally depends on a number of factors including flower species (da Rocha‐Filho et al. 2018; Nascimento et al. 2020; Threlfall et al. 2015), floral richness (Birdshire et al. 2020; Lerman et al. 2018; Quistberg et al. 2016; Threlfall et al. 2015), floral abundance (da Rocha‐Filho et al. 2018; Prendergast 2023b; Quistberg et al. 2016; Salisbury et al. 2015; Twerd and Banaszak‐Cibicka 2019), amount of impervious surfaces (Geslin et al. 2016; Sivakoff et al. 2018; Threlfall et al. 2015), local and landscape characteristics (Leonard et al. 2018; Quistberg et al. 2016; Senapathi et al. 2017; Sivakoff et al. 2018), bare ground cover (da Rocha‐Filho et al. 2018; Quistberg et al. 2016), grass cover (Lerman et al. 2018; Quistberg et al. 2016; Twerd and Banaszak‐Cibicka 2019), availability of nesting materials (Threlfall et al. 2015), history of the landscape (Threlfall et al. 2015; Twerd and Banaszak‐Cibicka 2019), urbanisation gradient (Birdshire et al. 2020; Geslin et al. 2016; Normandin et al. 2017; Sing et al. 2016; Threlfall et al. 2015), honeybee population abundance and density (Elbgami et al. 2014; Paini and Roberts 2005; Valido et al. 2019), road proximity (Leonard et al. 2018), disturbance (Winfree et al. 2009), and level of habitat fragmentation (Winfree et al. 2009), among other factors (Figure 2). Many of these influences have previously been examined only in non‐Australian bees, and whether the results are transferrable to an Australian context is relatively unknown. Some management practices developed in the Northern Hemisphere are inappropriate for Australian flora (Hopper 2009). For example, nitrogen and phosphorus nutrient additions to soil tend to promote plant growth in the Northern Hemisphere, but on the old, climatically‐buffered, infertile landscapes over much of Australia, nutrient additions can favour invasive weeds over native plants, or even lead to toxicity (Hopper 2009).

### 2.2 What Threats Are There?

Threats to native bees include habitat loss/fragmentation, urbanisation, agricultural intensification, climate change, altered fire regimes, pesticides, air pollution, pests and disease, and the introduction of non‐native plant and bee species (Figure 2) (Batley and Hogendoorn 2009; Goulson 2019; Heard 2016; Sands 2018; Schwarz and Hogendoorn 1999). However, the severity of these threats likely varies by bee guild (De Palma et al. 2015; Hofmann et al. 2019; Jauker et al. 2013; Persson et al. 2015; Williams et al. 2010), and with the lack of monitoring and knowledge of basic life‐history traits of the majority of native bee species, perhaps the biggest threat is a lack of species‐specific information about Australian bees (Batley and Hogendoorn 2009; Houston 2018). Figure 2 provides a schematic representation of the multiple and interacting threats that bees face from major environmental and anthropogenic changes, including climate change, land‐clearing, environmental and landscape factors, introduction of exotic species, agricultural intensification and urbanisation. Figure 2 further breaks down these major threats into related events and changes that can influence the survival and fitness of threatened bee species. The specific impacts of these threats are discussed in Sections 3 and 4.

### 2.3 Threats to Currently Listed Threatened Bee Species

The threats identified for the three species listed as critically endangered in Australia, *L. douglasiellus* , *N. simplicior* and *H. douglasi* , include habitat destruction, disturbance, fire and invasive species. Threats specific to *L. douglasiellus* include land clearing which has likely destroyed suitable habitat, mowing and other habitat disturbances (Department of Sustainability, Environment, Water, Population and Communities 2013), inappropriate fire regimes allowing weeds to take over areas with native vegetation (Threatened Species Scientific Committee 2013; Department of Sustainability, Environment, Water, Population and Communities 2013), and potentially competition from the introduced honeybee for floral resources (Houston 2018). A range of threats could impact the small area which this species occupies. The main threats to *N. simplicior* include bushland clearing for residential purposes, rural and industrial development, draining of winter‐wet depressions, and fire. *Neopasiphae simplicior* may also be at threat from competition with introduced honeybees (Department of the Environment, Water, Heritage and the Arts 2008). *Hesperocolletes douglasi* is potentially threatened by habitat clearing, habitat degradation, fire, pesticides, herbicides and competition with introduced honeybees (Threatened Species Scientific Committee 2019).

*Xylocopa aerata* (family: Apidae) became locally extinct on mainland South Australia and Victoria due to habitat fragmentation and fire (Glatz et al. 2015; Hogendoorn, Glatz, and Leijs 2020; Hogendoorn, Leijs, and Glatz 2020; Santos et al. 2020), and is being considered for inclusion on the new Flora and Fauna Guarantee Act Threatened List in Victoria. *Xylocopa aerata* now occurs only on Kangaroo Island in South Australia, around Sydney, and the Great Dividing Range in New South Wales. Following declines from bushfires in 2007 on Kangaroo Island, South Australia, in 2013, artificial nesting stalks were provided which enabled successful reproduction of almost 300 female bees (Hogendoorn, Glatz, and Leijs 2020). However, recovery has been hampered by climate change, and bushfires in January 2020 destroyed all 150+ artificial nests installed on Kangaroo Island, leaving only 5% of habitat intact, as well as burning much of their natural habitat in New South Wales. The major threats to this species include large, frequent fires, which destroy their Banksia nesting habitats, land clearing and climate change (Glatz et al. 2015).

## 3 Negative Consequences Associated With Anthropogenic Changes

Anthropogenic modifications of the environment or ecological processes can impinge on the fitness of native bee species. Acknowledging that there has been sparse research into many of these potential threatening processes in how they affect native bees, below we detail some of the threats and their studied and/or predicted impacts in Australia.

### 3.1 Land‐Clearing and Habitat Fragmentation or Loss

Habitat loss is recognised as the leading cause of bee declines worldwide (Brown and Paxton 2009). Land‐clearing destroys floral and nesting resources and can fragment populations. Unfortunately, the impact of historic landscape changes—such as habitat loss, vegetation structural and composition changes, and replacement of original vegetation due to agricultural and urban development in Australia on species distribution and populations will never be known due to a deficiency of data before the changes occurred (Batley and Hogendoorn 2009). However, we can make informed hypotheses based on recent studies examining habitat associations of native bee assemblages in areas subjected to various degrees of anthropogenic influences.

### 3.2 Urbanisation

Urbanisation results in an increase in impervious surfaces, temperature increases, loss of habitat, pollution, greater non‐native flora and fauna, and an increase in pesticides and herbicides (Faeth et al. 2012), all of which likely adversely impact native bees. General consequences of urbanisation on bees include lower flower visitation rates, low species richness, loss of rare species and homogenisation of species pools (Baldock 2020). However, research has been biased towards Northern Hemisphere systems, and specific effects on Australian bees are relatively unstudied (Prendergast et al. 2022b).

A few studies have been conducted in rapidly expanding large cities on the east coast of Australia—Melbourne, Brisbane, Sunshine Coast, Bundaberg and Sydney (Kaluza et al. 2016; Makinson et al. 2017; Shrestha et al. 2021; Threlfall et al. 2015). In Melbourne, native bees preferred less urbanised habitat locations (Shrestha et al. 2021; Threlfall et al. 2015). In contrast, bee hotel uptake in Sydney appeared unaffected by urbanisation (Makinson et al. 2017), and stingless bee ( *T. carbonaria* ) foraging in Queensland benefitted from urbanisation (Kaluza et al. 2016).

Research on the west coast of Australia has found more consistent adverse impacts of urbanisation. Native vegetation habitat was superior in supporting native bee assemblages (Prendergast 2020a, 2020b; Prendergast et al. 2020), and fitness of cavity‐nesting bees (Prendergast 2023a). In addition, when comparing pollination networks between native vegetation (bushland) remnants and residential gardens in urban areas, it was found that generalist species, including the non‐native honeybee ( *A. mellifera* ), and to a lesser extent, native *Amegilla*, *Exoneura*, *Lasioglossum* and *Homalictus* species, were associated with urban residential gardens, while the more specialised native Euryglossinae, *Leioproctus* and *Megachile* were positively associated with the remnant bushland habitat (Prendergast and Ollerton 2021). Additionally, research on a single species in this region, *Megachile ignita*, has also revealed a strong dependency on native resources in remnant bushland in urban areas (Prendergast 2018).

While these studies reveal the impact of urbanisation at local levels (examining specific cities or regions), larger evaluations of the effects of urbanisation on bees are required as urban sprawl increases.

### 3.3 Introduced Bees

The introduction of non‐native flora and fauna is, behind land‐clearing, identified as a major threat to pollinators (Brown and Paxton 2009). Australia has very few non‐native bees; however, the European honeybee, *A. mellifera* , is currently a dominant species in many assemblages, with evidence, albeit conflicting and to varying degrees of strength, to suggest that this introduced bee can exert a negative impact on native bees, as well as pollination networks, primarily through resource competition (Gross 2001; Paton 1993; Prendergast, Dixon, and Bateman 2021). Reviews on the impact of honeybees on native bees in Australia have revealed conflicting effects, including no strong evidence for negative impacts (Paini 2004) as well as negative significant and non‐significant impacts (Prendergast et al. 2022a).

Of the research conducted to date, the introduction of honeybee hives has been found to decrease native bees visiting flowers (Pyke 1990), and the presence of honeybees has been found to reduce the fecundity of the native bee, *Hylaeus alcyoneus* (Paini and Roberts 2005). Resource competition with honeybees can also not only reduce cell provisioning and increase mortality, but have more subtle fitness impacts such as a male biased sex ratio (Prendergast, Murphy, et al. 2025). Whilst these and other studies have shown negative impacts on native bees (Bailey 1994; Gross 2001; Sugden and Pyke 1991), there have also been studies finding no overall impact on some native bee species (Horskins and Turner 1999; Paini et al. 2005), or a mixed effect (Paini 2004). Recent research on the interactions between honeybees and native bees found that the impact of honeybees was complex depending on floral resources, habitat type and bee guild (Prendergast, Dixon, and Bateman 2021). Negative associations were more likely under high total floral resource levels, earlier in the day, and when niche overlap was high. Competition was exacerbated in residential gardens, and taxa that were most vulnerable were specialists (subfamily: Hylaeinae) with high niche overlap, and larger bodied bees (Prendergast, Dixon, and Bateman 2021). Honeybees may also negatively impact pollination of native flora compared to native bees (Gross 2001), and disrupt pollination networks (Prendergast, Dixon, and Bateman 2021).

There is evidence that *A. mellifera* could transfer pathogens or disease to native bees, both through the recent invasion of the Varroa mite in Australia (Chapman et al. 2023) and other transfer mechanisms, such as through contact with flowers (Purkiss and Lach 2019). The limited research in Australia indicates pathogen spillover can occur with harmful consequences (Purkiss and Lach 2019), whilst there is a larger body of global evidence supporting disease and pathogen spillover as an indirect threat that *A. mellifera* can pose to native bee communities (Dalmon et al. 2021; Fleites‐Ayil et al. 2023; Nanetti et al. 2021; Tehel et al. 2016).

### 3.4 Introduced Flora

As with introduced bees, introduced flora may also exert negative impacts on native bees (Stout and Morales 2009). These negative impacts can occur through exotic flora outcompeting native, preferred flora (exotic grasses may be particularly problematic given they provide limited resources) (Davies 2011); or as introduced plant species may favour introduced competitors like *A. mellifera* (Aizen et al. 2008, 2014; Morales and Aizen 2002, 2006). There can also be synergistic interactions between exotic plants and exotic bees (Goulson and Derwent 2004; Stokes et al. 2006; Stout et al. 2002).

Given that native plants tend to be preferred by native bees (Corbet et al. 2001; Elliott et al. 2021; Howard and Symonds 2023; Pardee and Philpott 2014; Prendergast 2023b; Prendergast and Ollerton 2021), habitats dominated by introduced flora often harbour fewer bees and, by reducing the proportion of native, preferred plants in any area, this likely reduces foraging efficiency (Prendergast and Ollerton 2021).

The potential threat of non‐native flora is particularly of concern for oligolectic bee taxa, which rely on a narrow taxonomic range of flowers (Praz et al. 2008; Sands 2018). An example is Euryglossinae, the most species‐rich subfamily, where the majority of species are oligolectic (Houston 2018, 2000).

Non‐native flora may provide resources for generalist bees (Kaluza 2017), especially when native flora are scarce, such as at the start or end of a species' foraging season (Mach and Potter 2018). Recent work has shown that the generalist native Australian bee, *Lasioglossum lanarium* , prefers non‐native weed flowers compared to a native angiosperm when competition with honeybees and other insects is removed from the decision‐making process (Howard and Symonds 2023). Such findings likely depend upon the bee and plant species in question, and given the long co‐evolutionary history of Australia's floral and bee communities, non‐native flora might not satisfy all the nutritional demands of Australian bees (Parreño et al. 2022), particularly Australia's oligolectic bees which specialise on pollen from native plant species (Armstrong 1979; Cane and Sipes 2006; Hingston 1999; Iwasaki and Hogendoorn 2023; Williamson et al. 2025).

### 3.5 Pesticide‐Use

The negative impacts of the most widely used pesticides, neonicotinoids (Douglas and Tooker 2015; Sparks 2013), on important pollinators such as bees have become a highly recognised issue in the debates regarding insect decline and food security (Goulson et al. 2015). While there are limited studies examining the impact of pesticides on native Australian bees (Batley and Hogendoorn 2009; Hereward et al. 2026), research and case studies from across the globe can inform us of the impacts on bees in general.

Studies show that neonicotinoids can cause sublethal effects in different bee species. These impacts include cognitive (Decourtye et al. 2004; Goñalons and Farina 2015; Wright et al. 2015) and navigational (e.g., homing ability) (Fischer et al. 2014; Henry et al. 2012) impairments in *A. mellifera* . Neonicotinoid exposure results in reduced colony growth and reproduction (Rundlöf et al. 2015; Whitehorn et al. 2012) in *B. terrestris* , and sociality (Crall et al. 2018) and nursing impairments in *B. impatiens* (Crall et al. 2018). These pesticides also cause foraging impairments (Feltham et al. 2014; Gill and Raine 2014; Gill et al. 2012; Muth and Leonard 2019; Stanley et al. 2016) in *B. terrestris* and *B. impatiens* , including a reduction in crop pollination services by *B. terrestris* (Stanley et al. 2015). While much less is known about the impacts on non‐eusocial wild bees, neonicotinoids do result in a reduction in wild bee density (Rundlöf et al. 2015), an solitary bee nesting (Chan and Raine 2021; Rundlöf et al. 2015), reproduction (Chan and Raine 2021) and pollen collection (Chan and Raine 2021). In contrast, there are some studies showing no impacts of neonicotinoids on bees in certain cases (Manning 2018; Strobl et al. 2021).

The current assessments of pesticide risks on bees have largely been with *A. mellifera* despite most of the world's 20,000 bee species being solitary (Sgolastra et al. 2019). A recent review argues that due to differences between life history traits in solitary bees compared to the eusocial honeybee, there are likely to be significant differences in routes and levels of pesticide exposure (Sgolastra et al. 2019). Thus, our current understanding of how bees are impacted by pesticides is poor and largely limited to a single species.

It is important to examine the impact of pesticides on native Australian bees, as Australia is still using many common neonicotinoids now deemed too dangerous to use in other countries due to their impact on bees (e.g., Imidacloprid) (Gross 2013; Howard and Ren 2024).

### 3.6 Climate Change, Floods and Fires

Climate change is a current and ongoing threat to biodiversity (Bellard et al. 2012). Climate change may impact bees by causing increased temperatures, shifts in phenology, altered distributions, altered nectar and pollen chemistry, and an increase in extreme weather events, including damaging bushfires and floods (Batley and Hogendoorn 2009; Forrest 2015; Herrera 2020, 2024; Herrera, Núñez, Aguado, and Alonso 2023; Herrera, Núñez, Valverde, and Alonso 2023; Hoover et al. 2012; Ogilvie et al. 2017; Soroye et al. 2020; Stemkovski et al. 2020; Ziska et al. 2016). Additionally, a rise in atmospheric carbon dioxide (CO_2_) can reduce the protein concentration of pollen, an essential dietary requirement for bees, although long‐term impacts of this on bees need to be further explored (for example: (Hoover et al. 2012; Lake and Hughes 1999; Ziska et al. 2016)). Floods are known to be a threat to ground‐nesting bees (Fellendorf et al. 2004) and are common in northern Australia while fires have mixed positive and negative effects on both cavity and ground‐nesting bees (Cane and Neff 2011; Potts et al. 2005), as well as specialist and generalist bees (Peralta et al. 2017).

Fire can have complex influences on bees, ranging from positive to negative, depending on the extent, severity and bee guild (Prendergast et al. 2025). Until recently, there had been little research into the impacts of fire on native bees and other Australian invertebrates (Saunders et al. 2021). Research in other regions has produced mixed results, from positive (e.g., (Galbraith et al. 2019)) to negative (e.g., (Potts et al. 2001)). Recent modelling of impacts from the 2019 and 2020 bushfires on Australian native bees found that nine species were eligible to be listed as Vulnerable and two as Endangered due to the fires (Dorey et al. 2021). Further on‐the‐ground surveys are required, and furthermore, two‐thirds of native bee species were unable to be modelled as there was insufficient collection information (Dorey et al. 2021). Under climate change, Australia has experienced, and can expect, more damaging and frequent bushfires (Jones et al. 2013; Lucas et al. 2007). Bushfires can cause damage to and loss of essential nesting habitats and materials for native bees. As discussed above, the fires on Kangaroo Island and in New South Wales in 2020 destroyed suitable habitats and artificial nests designed for the conservation of *X. aerata* (Hogendoorn, Leijs, and Glatz 2020). The extinction of *X. aerata* in Victoria (Victoria State Government, Department of Environment, Land, Water and Planning 2020) is suspected to have occurred through a combination of land‐clearing and burning (Glatz et al. 2015). Fires are also a threat to the Critically Engendered *L. douglasiellus* , *N. simplicior* and *H. douglasi* (Department of Sustainability, Environment, Water, Population and Communities 2013; Department of the Environment, Water, Heritage and the Arts 2008; Threatened Species Scientific Committee 2019). Despite the threats posed to bees, fire can also have positive impacts, see Section 4 below.

## 4 Positive Consequences for Australian Native Bees Associated With Anthropogenic Changes

Anthropogenic activities, whilst having negative impacts, can also have beneficial outcomes for wild bees. A number of studies outside of Australia have found that bees perform well in cities (Banaszak‐Cibicka and Żmihorski 2020; Hall et al. 2017; Nascimento et al. 2020; Samuelson et al. 2018) and that urbanisation has positive effects on bees such as higher species richness (Baldock et al. 2015; Theodorou et al. 2017) and increased reproductive success in cities compared to farmland (Samuelson et al. 2018). A recent study across urban gradients in Africa found that urbanisation did not affect bee abundance or diversity; however, it had a negative effect on the abundance of wasps and beetles (Guenat et al. 2019).

In Australia, there is scant evidence of positive effects of anthropogenic change on native bees. This lack of data may be due to the scarcity of research on this topic. It is also likely due to the specialised co‐evolution between plants and bees, which have been radically altered by anthropogenic influences, compared with Northern Hemisphere landscapes that have been subjected to longer periods of modern human disturbance. Impacts of climate change on Australian bees include some beneficial impacts to certain species. For example, projected climate change models expect an increase in the distribution of the native Australian bee, *Ceratina australensis* , which already has a wide distribution. This research shows that climate change may have potential positive effects on the ranges of other native Australian bees as well (Dew et al. 2019). Some Australian species also appear to do well in anthropogenic environments. Some examples include (1) *Leioproctus plumosus* , which has frequently been recorded nesting in residential gardens (Prendergast and Ollerton 2021), (2) the eastern Australian bee, *Hylaeus nubilosus* , which has expanded its distribution due to unintentional human‐mediated transport and has successfully established in Western Australia (Prendergast 2017), (3) *Hylaeus ruficeps* *kalamundae*, which has adapted to nesting in anthropogenic materials in abundances never seen before in nature (Prendergast 2020b), (4) Meliponini bees, especially *T. carbonaria* , which have increased exponentially under ‘sugarbag’ bee keeping and husbandry (Halcroft et al. 2013; Heard 2016), (5) *T. carbonaria* , which has also been found to forage more successfully in urban environments compared with agricultural macadamia plantations and even natural vegetated landscape types (Leonhardt et al. 2016), and (6) the phototactic response (attraction to light) of native Australian bees, predominantly *Lasioglossum*, appears to be unaffected by the urbanisation level of the individual's habitat (Webster et al. 2025).

While fires are known to pose a threat to bees (Cane and Neff 2011), they also can provide opportunities and resources. Newly burnt areas often host increased species richness (Galbraith et al. 2019; Peralta et al. 2017; Potts et al. 2005; Simanonok and Burkle 2019) and bee abundance (Galbraith et al. 2019; Grundel et al. 2010), which declines as the time since a fire has occurred increases. One reason for this may be that fires allow more bare ground to be exposed, thus ground‐nesting habitat availability increases, which decreases as vegetation regenerates (Potts et al. 2005).

## 5 How to Assess and Continue Monitoring the Situation

Suggestions for policies, management and public engagement for insect conservation in Australia have been detailed in recent years (Pyke et al. 2023; Sands 2018; Taylor et al. 2018), however these approaches still acknowledge that native pollinators, including native bees, require further research to determine whether they and their breeding habitats are threatened (Sands 2018). Below we outline what important information is missing (Section 5.1), how we can monitor native bee populations (Sections 5.2 and 5.3), and pathways to assessing the impact of potential threats early (Section 5.3).

### 5.1 What Is Missing?

As discussed above, the paucity of information on native Australian bees is one of their biggest threats (Batley and Hogendoorn 2009; Dyer et al. 2023; Sands 2018; Taylor et al. 2018). Their biology, distribution, breeding behaviour, habitat requirements and crop pollination abilities are understudied (Batley and Hogendoorn 2009; Houston 2018). Contributions to this problem are suggested to include a lack of taxonomic knowledge (Hoh 2020), poor biological knowledge, limited funds and expertise, a lack of education, a lack of awareness, and a scarcity of taxonomists and insect ecologists (Hoh 2020; Sands 2018; Taylor et al. 2018). Furthermore, very few Australian native bees have been assessed for their conservation status due to this scarcity of knowledge (Houston 2018). Despite these limiting factors, many researchers have and continue to provide valuable experimental data and observations on native Australian bee biology, behaviour, distribution, breeding behaviour, habitat requirements, genetics, thermal tolerances and crop pollination abilities (for examples see: (Brown et al. 2020; Bull and Schwarz 1997; da Silva et al. 2026; Dorey et al. 2020; Elmer et al. 2025; Fernandes et al. 2025; Hall 2018; Hall et al. 2019; Heard 1994; Howard, Prendergast, et al. 2021; Hurst et al. 1997; Joyce and Schwarz 2006, 2007; Kayaalp and Schwarz 2008; Kreider et al. 2025; Langer et al. 2006; Law et al. 2025; Lentini et al. 2012; Neville et al. 1998; Prendergast 2018; Rehan et al. 2010; Schwarz and O'Keefe 1991; Schwarz et al. 1996; Seeman et al. 2025; Tierney et al. 1997)).

There is limited behavioural data on native bees, especially compared to introduced eusocial species such as *A. mellifera* and *B. terrestris* . The lack of information makes it difficult to determine whether behaviour, such as foraging strategies and choices, floral trait preferences, or personality traits (e.g., boldness; aggression) change depending on environmental disturbance by humans, increased introduced flora, or competition with introduced bee fauna. Learning, memory, and decision‐making are key factors in determining reponses to environmental changes, as these behaviours underpin behavioural plasticity, allowing animals to adapt to change. In terms of controlled behavioural testing, the eusocial native bee, *T. carbonaria* (previously *Trigona carbonaria* ), has been studied for its colour preferences (Dyer et al. 2019; Dyer, Boyd‐Gerny, et al. 2016; Forster et al. 2023), nectar temperature preference (Norgate et al. 2010), flower constancy behaviour (White et al. 2001), learning capacity (Mourmourakis and Barron 2025), flight performance (da Silva et al. 2025; Smith et al. 2017), pollen transport (Brennan et al. 2026) and flower detection (Dyer, Streinzer, and Garcia 2016). Recently, two species of native halictid bees (family: Halictidae), *L. lanarium* and *L*. (*Parasphecodes*) sp., have been tested for their colour preferences under controlled laboratory conditions, which has important implications for foraging choices by these native species (Howard, Garcia, and Dyer 2021). *L. lanarium* has also recently demonstrated the ability to learn to discriminate between colours and thus avoid certain stimulus colours associated with a predation event (Howard 2021). Considering Australia is home to an estimated 2000 or more species of native bees (the vast majority of which are solitary), an expansion of this research topic is essential to assess, monitor and act to support Australia's native bees.

An important, yet time and economically costly, aspect of better understanding Australia's native bees is longer‐term studies (Neave et al. 2020). Some of the most useful data on species declines and losses come from long‐term data collection (Magurran et al. 2010). Long‐term data and monitoring allow us to better understand the current environment, fluctuations and finally determine whether there is an overall trend of increasing, decreasing or stability in bee populations and what may be causing changes. The value of these ideas is discussed further below in Sections 5.2 and 5.3.

### 5.2 How We Monitor Bee Populations

We can monitor bees in several ways, including surveys and citizen/community science. Recently a review, supplemented by an empirical study conducted in Western Australia, compared different traditional sampling techniques to determine which one was most effective for Australian native bees (Prendergast et al. 2020). The study compared observational records, targeted netting, mobile gardens, pan traps (blue and yellow), vane traps (blue and yellow) and trap‐nests for their effectiveness at capturing bee species. Similar to other studies, vane and pan traps were found to be an ineffective way of collecting certain bee specimens of specific species (Howard, Ratnayake, et al. 2021; Popic et al. 2013; Prendergast et al. 2020; Shrestha et al. 2019, 2021), however, they can be useful for certain data collection (Howard, Ratnayake, et al. 2021). Observational counts appear to record the highest number of bees; however, sampling where bees are collected is necessary to perform finer taxonomic classification. A review of studies conducted both in Australia as well as elsewhere suggests that sweep netting appears to be an efficient way to measure bee abundance as well as classify species, and unlike passive methods provides crucial information about foraging preferences (Prendergast et al. 2020). The consensus is that different methods capture different species and thus may be suited to different experimental questions and taxa depending on the research question (see Prendergast et al. (2020) and references therein). When resources, funding and access to equipment permit, multiple survey methods are preferable.

There are a few community/citizen science projects which promote public involvement in pollinator data collection in Australia. Public engagement has been critical to the recent popularisation of the honeybee in the past decade (Dyer et al. 2020; Howard and Dyer 2020; Smith and Saunders 2016), and pollinators in general (Howard and Dyer 2020). Engaging the public in insect conservation may be a critical step in supporting pollinators (Hall and Martins 2020; Harvey et al. 2020; Saunders et al. 2018; Wilson et al. 2017), and more efforts are needed given large gaps in the public's understanding of Australian bee biodiversity (Prendergast 2025). People are also able to contribute to data collection in places such as iNaturalist (https://www.inaturalist.org/), not to mention a number of bee and pollinator projects in other countries [e.g., see (Mason and Arathi 2019)]. These community science outreach projects allow the public to participate in data collection, permit a large amount of data to be collected, help to inform the community about pollinators, and can engage the public in research and conservation efforts. For example, the Wild Pollinator Count, which has been running since 2015 (Saunders 2018), had over 2000 submissions in 2020 for its autumn count with over 20,000 insects being observed during the week‐long period of counting. A recent examination of social media posts allowed mapping of a native bee genus (*Amegilla*) and non‐native bee species ( *A. mellifera* ) (ElQadi et al. 2017) and suggests further work into the value of social media and community engagement for species distribution mapping. Location‐tagged social media images and iNaturalist records can be a valuable source of occurrence data to supplement insect surveys, particularly for understudied species with a paucity of expert‐verified distribution information (Chowdhury et al. 2026; Moore et al. 2024). There are numerous limitations in citizen science projects, including for the majority of species, the inability to identify them to species level, biased collections towards areas or flora people are more likely to observe, and biases towards easily identifiable, popular taxa. Designing citizen science projects that enable robust conclusions is a challenge (Prendergast, Vanderstock, et al. 2022). Recent work on the citizen science project, Native Bee Watch, has suggested that this method is valuable for monitoring bees in an urban area depending on the level of volunteer engagement and training (Mason and Arathi 2019) and while time‐consuming, training volunteers can provide important for monitoring opportunities and improve data accuracy (Garratt et al. 2019).

### 5.3 Monitoring Impacts

Ultimately, long‐term surveys are necessary to monitor changes in species richness, distribution, morphology, behaviour and population densities. While these surveys can be costly and time‐consuming, the benefit the data can provide is worth the effort. Considering significant insect declines have been observed in long‐term studies (Hallmann et al. 2017; Lister and Garcia 2018), surveys such as these should be supported in Australia (Neave et al. 2020), particularly because of the unique bee fauna (Houston 2018; Michener 2007).

Long‐term data can also help to monitor behavioural, morphological and physiological changes in response to threats. For example, with rising temperatures in cities, leaf‐cutter ants (*Atta sexdens rubropilosa*) have become more tolerant to heat (Angilletta Jr. et al. 2007). The use of museum specimens compared across time has shown that social wasps (*Dolichovespula sylvestris*) are decreasing in size in response to climate change (Polidori et al. 2020). Similarly, urbanisation appears to be correlated with wing shape asymmetry in *A. mellifera* , signifying stress related to landscape traits such as road proximity, anthropogenic land area, vegetation area (Leonard et al. 2018) and high human disturbance (Nunes et al. 2015). With an examination of behavioural, morphological, and physiological changes and/or adaptation to threats over time, we can begin to understand how native Australian bee species are reacting to the Anthropocene.

Another valuable resource is modelling bee distributions in response to potential threats. Climate change models with native bee distribution projections have been performed with interesting results, as discussed above (Dew et al. 2019; Santos et al. 2020). Predicting the occurrence and range of threats such as urbanisation, habitat fragmentation and climate change, among other threats, could allow us to anticipate impacts on native species and act before threats become unmanageable.

In 2020, a framework was published detailing how to move forward in insect conservation and recovery (Harvey et al. 2020). This plan suggests action at immediate, short, intermediate and long‐term time scales. Immediate and short‐term action proposes using insect‐friendly techniques in farming habitat management and urban planning/development as well as assessing the conservation status of insects to determine priority species, areas and issues to be addressed. Intermediate action involves the use of existing data on insect diversity to determine the current status of insect populations and enable us to compare this information to new research. New research would involve determining the impact of different anthropogenic stressors on insect declines and increasing exploratory research. Long‐term action is suggested to include developing partnerships with public and private stakeholders to restore, protect and create suitable insect habitats and mitigate threats. Finally, a global approach to insect monitoring is vital to determining the rate and severity of insect declines and recovery (Harvey et al. 2020).

## 6 A Look at the Diversity and Conservation Status of Native Australian Bees 15 Years on

As highlighted in the Introduction, Batley and Hogendoorn (2009) set the stage for raising awareness about the diversity and conservation status of Australian native bees. Fifteen years on, our review asks, ‘What has changed?’ There has been an increase in the number of described species by ~76 species; however, when we break this down by family it is evident that there have also been many synonymisations, evidencing the need for further robust taxonomic work. New taxonomic methods may aid in streamlining the identification process, with the potential for non‐lethal taxonomic methods, but taxonomic expertise remains a critical component in wild bee conservation (Moore et al. 2026). Fifteen years ago, Batley and Hogendoorn (2009) estimated that there were 500 undescribed species; thus, it is clear that there is still much work to be done. Batley and Hogendoorn (2009) conclude that ‘the major constraint on bee conservation, however, is the severe shortage of information and expertise’, and this unfortunately still seems to be the case as evidenced by our current review.

Batley and Hogendoorn (2009) provided two possible strategies for conserving existing bee species including the development of commercial applications, and the enhancement of public interest. Despite Amegilla and Xylcopa being identified for use as potential for commhoneercial pollinators, especially of buzz‐pollinated crops, research is still ongoing for *Amegilla*, and no research to our knowledge has involved *Xylocopa*. There has been much attention towards advancing stingless bees as commercial pollinators, with both research and commercial operations (e.g., Greco et al. 2011; Halcroft et al. 2013; Heard 2016; Meléndez Ramírez et al. 2018; Subasinghe Arachchige et al. 2022). Whilst there has been a large investment into exploring crop pollination capacity (Bernauer et al. 2022; Hogendoorn and Keller 2011, 2012; Hogendoorn et al. 2021; Nacko et al. 2022; Reynolds and Robinson 2022), few recommendations have been actioned. Pollination for non‐crop services is in its infancy. When it comes to commercial applications, the focus is largely on sugarbag bees. Furthermore, despite an Australian Native Bee Strategic RD&E Plan (2022–2027) being produced, few recommendations outside those involving sugarbag bees have been actioned, and there appears to be no plan to revise and update it.

When it comes to developing public interest, much progress has been made, but there remain many challenges. The Australian Pollinator Week and Australian World Bee Day celebrations appear to be gaining traction, however they still largely feature the introduced European honey bee. Concerningly, whilst striking and identifiable taxa, such as blue‐banded *Amegilla*, are becoming popular among the public, few Australians can name other species (Prendergast 2025). Encouragingly more and more councils are beginning to promote native bees. In the state of New South Wales, both Ku‐ring‐gai Council and Cumberland Council operate programmes where residents can foster hives of native stingless bees. In Melbourne, a grass‐roots community effort is installing pollinator gardens, and the City of Melbourne released a public educational book of insects that features native bees titled, ‘Little Things That Run the City’ (Cranney et al. 2017). In the state of Western Australia, the City of Bayswater, Melville, Perth and Cockburn are all investing in native bee monitoring and greening strategies.

We have observed increased knowledge regarding native bee behaviour, a better understanding of their responses to environmental changes, increased community science contributions, and improvements in their use as commercial pollinators. However, as this review emphasises, Australian research, industry and communities still have a long road ahead as we work towards comprehensive and knowledge‐based conservation, management policies and support for native bees.

## 7 Australian Native Bees in a Global Context: Lessons and Insights

The conservation of Australian bees has major relevance to a global audience. First, virtually all of the described and estimated species are endemic, with entire families and subfamilies occurring only on this continent (Houston 2018). We stand to lose a major, unique component of bee diversity if we fail to protect Australian bee fauna. The evolution of plants and pollinators in isolation, together with the diverse landscapes and flora, offer major insights into a host of scientific topics such as introduced species and resource competition (Prendergast, Dixon, and Bateman 2021), speciation (Armbruster et al. 1994), plant reward systems (Stone et al. 2003), the evolution of sociality (Schwarz et al. 1998) and mating tactics (Alcock 1997). Australia is a ‘megadiverse country’ (McDonald et al. 2015), with two of the 35 global biodiversity hotspots (Myers et al. 2000), and pollinators are major contributors to the high biodiversity of these locations. Like with bees, Australia's mammalian fauna is largely endemic (Houston 2018). Unlike with bees, we have a greater understanding of what has been lost and what is at risk: Australia has the worst extinction record of mammalian fauna of any country over the last two centuries, and today 21% are listed as threatened (Woinarski et al. 2015). This dire situation may very well hold for bees, but we currently lack the data to know.

Australia should have the resources to tackle the issues we outline above. The threats and issues bees face, and how we address these challenges are not idiosyncratic to Australia. As such, our recommendations for advancing the knowledge and conservation of Australian bees, and threats to bee conservation, provide a roadmap for bees in other biodiverse, yet under‐studied, regions.

## 8 Conclusion

The major threat to Australia's native bees remains the lack of information, knowledge, and expertise (Batley and Hogendoorn 2009). We propose that urgent action is taken to develop a better understanding of native bee behaviour, distribution, conservation status, foraging strategies, flower preferences, and pollination abilities, especially in response to the threats outlined in this review. In particular, we propose a need for longer‐term studies and surveys using robust methodologies, a continuation of the work through Howard community science, and a focus on examining behaviour and foraging patterns of native bees. Only then can we protect native bee biodiversity in the face of anthropogenic and environmental change.

## Author Contributions

**Scarlett R. Howard:** conceptualization (lead), funding acquisition (lead), investigation (lead), methodology (equal), project administration (equal), resources (equal), writing – original draft (lead), writing – review and editing (equal). **Kit S. Prendergast:** conceptualization (equal), funding acquisition (equal), investigation (equal), methodology (equal), resources (equal), writing – review and editing (equal).

## Funding

This work was supported by the Hermon Slade Foundation: HSF21076, ARC Discovery Early Career Research Award (DECRA): DE230101556, and funding from Deakin University's Alfred Deakin Postdoctoral Research Fellowship.

## Conflicts of Interest

The authors declare no conflicts of interest.

## Data Availability

No new data was produced for this article.
